# Attitudes and practices of Sudan’s health sector partners towards strengthening and utilising the national health system components (2015–2022)

**DOI:** 10.1371/journal.pgph.0007039

**Published:** 2026-08-18

**Authors:** Huzeifa Aweesha, Anni-Maria Pulkki-Brännström, Anna-Karin Hurtig, Miguel San Sebastian

**Affiliations:** Department of Epidemiology and Global Health, Umeå University, Umeå, Sweden; Johns Hopkins University Bloomberg School of Public Health, UNITED STATES OF AMERICA

## Abstract

While the emphasis on strengthening and utilising a unified national system has gained momentum in discussions among health partners throughout the world, there are evident discrepancies between commitments and practice. Sudan presents an interesting case where the last decade has witnessed major political changes that have influenced health sector partnerships. This study investigates the shifts in attitudes and practices of Sudan’s health sector partners towards strengthening and utilising the national health system, specifically focusing on the components of financial management, procurement and supply, and health information. Utilising a qualitative case study design, data were collected through semi-structured interviews with key stakeholders from various humanitarian and developmental partners during two rounds (2015 and 2021/22). The interviews were analysed thematically, and situational analysis was applied with the partners’ stances displayed on positional maps. The findings revealed a shift from initial resistance to and prevention of system strengthening and utilisation to a more collaborative approach towards the system utilisation, influenced by political changes, and the need to adopt resilient approaches to respond to the volatile emergency context. Meanwhile, over time humanitarian partners have persisted to utilise their own systems because they have control over these systems or believe their systems function better. Moreovere, some respondents claimed that they are not mandated to support a unified system. Tension persists between utilising the system as prerequisite to strengthen it or ensuring the system is strengthened before using it. The study highlights the critical need for continuous monitoring and commitment to utilising the national system by all to ensure sustainability and enable timely improvement, especially in fragile contexts like Sudan. Flexible arrangements and guidance to encourage humanitarian partners to commit to abolishing parallel systems are crucial.

## Background

The emphasis on health systems and health systems strengthening has gained momentum during the era of the Millennium Development Goals. This has been facilitated by the emergence of partners supporting vertical disease-focused programmes, which have threatened to cause a deeper fragmentation of the health systems in low- and middle-income countries (LMICs) [1,2]. Given the mandate to support the health systems of LMICs, international partners are expected to help strengthen national system capacities, to respect national leadership and policies, and to utilise the national health system rather than their own systems [3,4]. The World Health Organization (WHO) defines health system strengthening as ‘the process of identifying and implementing the changes in policy and practice in a country’s health system, so that the country can respond better to its health and health system challenges’ [5]. Health sector donors and other international partners engage in a wide spectrum of activities and work to strengthen the health systems of LMICs. These often include improvements to infrastructure and governance capacities, human resources training, investments in national health information systems, and enhancing medical supply chains [3,6–8]. In this context, health system utilisation encompasses the use of unified public financial management procedures (budget execution, reporting, and auditing) that enhances the predictability and transparency of funding, a national health procurement and supply body, and a single health information system for all reporting [9]. In contrast to the agreed upon need for stronger health systems, it has been harder to transition to the utilisation of a national system exclusively because it means abandoning the standalone system(s), policies, and standard procedures of the agencies to reduce duplication and fragmentation [10].

Given that the number of partners continues to increase, the health sector throughout the world has embraced agreements emphasizing collaboration, in line with the Paris Declaration on aid effectiveness. In 2007, the International Health Partnership (IHP+) was established, aiming to improve coordination among health partners (donors, implementing agencies including the United Nations [UN] and international non-governmental organisations [INGOs], recipient governments, and civil society organisations [CSOs]) [11]. The IHP + led to the adoption of the ‘behaviours for development partners to cooperate better’ [9] and held monitoring rounds on the progress of effective development cooperation commitments and strategies. These behavioural commitments signed by health partners included supporting a single national health strategy; harmonising, aligning, and using national financial management, procurement and supply, and health information systems; recording all funds in the national budget; supporting south-to-south and triangular cooperation; and providing well-coordinated technical assistance. When the IHP+ transformed into the Universal Health Coverage partnership (UHC2030) in 2015, it kept the same core partnership strategies.

Strengthening and effectively using three core components of the national health system – (1) procurement and supply, (2) financial management, and (3) health information – aligns with the WHO’s recommendation to reinforce implementation levers as essential governance functions for achieving universal health coverage [12]. There is also a focus on the joint work of partners to decrease duplication and to enhance system efficiency. This emphasis on the strengthening and utilisation of these health system components has also been adopted by low-income countries that receive aid within their developed country health compacts [10]. While development partners have widely committed to supporting strengthening efforts and utilising national systems, there have been more successes and efforts documented on strengthening support. There are several reasons why utilisation of a national system can be problematic for partners: partners use of their own systems, practical (capacity and resource) and/or political constraints, and resistance to change. In their review of how recipient countries and global partners have performed in terms of their IHP+ commitments, Shorten et al. [10] found a lack of progress on the use of countries’ financial management and procurement and supply components. Moreover, there were issues regarding the integration of duplicative performance reporting frameworks into a single national health information system, despite signed commitments. Unfortunately, there has been a lack of studies on commitments to system strengthening and utilisation within the health sector since 2015, following the transition of the IHP+ to UHC2030. This transition led to the end of the IHP+ regular Effective Development Cooperation (EDC) monitoring rounds that facilitated comprehensive evaluation of EDC in the global health sector.

### The Sudanese health sector and Effective Development Cooperation

Following the separation of South Sudan in 2011, with the loss of oil and international support, Sudanese public services experienced a marked reduction in funding [13]. This was further worsened by the continuation of internal conflicts in Darfur, Blue Nile, and the Nuba Mountains, and by complicated international relations due to the investigation of the president by the International Criminal Court (ICC), leading to sanctions from several Western governments. Hence, the Sudan Federal Ministry of Health approached the IHP+ to help improve the country’s health sector partnerships and the capacity of its healthy system. Sudan joined the IHP+ in late 2011 and subsequently led internal deliberations with major health partners (donors, UN agencies, INGOs, and CSOs), resulting in the signing of Sudan Health Compact in 2014 [14].

The Sudanese health sector involves a diverse mix of humanitarian and development partners. Within this article we define humanitarian partners as entities whose primary mandate is to save lives and to immediately alleviate suffering during crises. Their work is guided by humanitarian principles (humanity, neutrality, impartiality, and independence) [15]. Humanitarian actors may include government bodies, donors, UN agencies, INGOs, and CSOs focusing on emergency relief. On the other hand, development partners are institutions – typically donors, agencies, development banks, and technical organisations – whose mandate is to support long-term social, economic, institutional, and governance development in partner countries. Their work focuses on system strengthening, resilience, capacity building, and achieving sustainable development [16]. Within the Sudan Health Compact, the health sector partners committed to harmonising and aligning with the national health system components while supporting their strengthening [14]. This commitment to strengthening was clarified as a way to ‘increase their support for strengthening the country’s structures, systems and procedures’. They further agreed to use the national health system as much as possible, thus building trust between partners and enabling capacity building, with financial management, procurement and supply, and health information as the focus of the compact. The compact further detailed commitments to ‘work together to simplify and harmonize the financial management’, ‘strengthen the Sudanese Government procurement and supply system and to ensure that all procurements and supplies gradually utilize it’. It mentioned that there would be one framework for monitoring and results in the country: the national health information system, where all data feeds. Furthermore, the health partners adopted the Humanitarian–Development–Peace Nexus (HDPNx) approach in 2017 as one of the sector’s strategic shifts. The approach plan was for joint analysis, planning, and programming, with humanitarian operations gradually being integrated into the national system [17].

There have been major contextual changes since then, with a revolution in 2019 overthrowing a dictatorship that had been in power for 30 years, during which time it had divided the country, committed human rights violations, and worsened global relations. For two years after the revolution, there was an influx of partners and hope for improved relations. However, a military coup in 2021 again led to strained international relations [18,19]. The challenges and needs of the health sector were magnified further by a nationwide armed conflict that broke out in April 2023 [20,21].

The limited evidence on partners’ progress in strengthening and utilising national health systems, coupled with the absence of monitoring mechanisms for these collaborative efforts, points to an urgent need for research on these topics. A deeper examination of how complex contextual factors influence partners’ adherence to their commitments is critical. Such a study can provide valuable insights into the dynamics of these partnerships and identify areas for improvement, ensuring that efforts to support national health systems truly contribute to strengthening them. Thus, this study investigated the attitudes and practices of Sudan’s health sector partners towards strengthening and utilising the national health system components of financial management, procurement and supply, and health information. Additionally, it explored how these attitudes and practices differed between 2015 and 2021/22, analysing these changes in the context of broader developments within Sudan during this period. It is worth noting that this study was conducted prior to the war in April 2023, so the findings do not account for the effects of the war.

## Methodology

### Study setting: The Sudanese health system

Sudan has a decentralised three-tier health system. The federal level is tasked with legislation, policy development, strategic planning, technical support for the two lower levels, the training and development of healthcare workers, overall monitoring, and external relations. The state level provides hospital services, employs workers, and has capacity-building functions. The localities are tasked with primary healthcare services, including environmental health issues, and the employment of assistant cadres. The Sudanese health system faces marked shortages and inadequate distribution of resources and capacities, with multiple recurrent outbreaks and economic, political, and conflict stressors leading to challenging health situations [22,23].

The Sudanese health sector has a variety of partners from different humanitarian and development groups, with humanitarian actors playing a dominant role [14,19,23]. Many donors support both humanitarian interventions and efforts to improve the health system (e.g., from the European Union, the European Civil Protection and Humanitarian Aid Operations [ECHO] for emergency financing and the European Commission Directorate-General for International Partnerships [DEVCO] for development cooperation). The World Bank and African Development Bank provide major investments for the national health system to build its capacities and to improve its resilience rather than financing emergency operations. UN agencies mainly provide support for development, although all UN agencies working on health have an emergency portfolio. The majority of INGOs and CSOs focus on the direct provision of services within humanitarian settings, and only a few have expanded to the development field following the adoption of the HDPNx. By 2015, the majority of spending on health in Sudan was out of pocket (70%), mainly on private-sector curative services, yet international support constitutes 75% of expenditure on primary healthcare services [24]. According to the WHO, this did not undergo a major change from 2015 to 2022 [23]. Additional details of the partner groups definitions, the various health sector partners within each group, and the scope of their work are available elsewhere [14,19,23].

Although the three health system components of interest – financial management, procurement and supply, and health information – have shown some progress in recent years, they continue to face challenges. A Joint Financial Management Assessment was conducted in 2016 to determine ‘whether or not the Public Financial Management (PFM) system of Sudan was adequate, and whether ongoing PFM reforms had strengthened it to a level that would be acceptable to development partners who supported investments in the health sector’ [25]. While the assessment concluded that the Government of Sudan had a robust statutory and regulatory framework for PFM, but the audit function was rudimentary, not based on risk, and lacked proper planning and trained personnel. Moreover, the external audit reports were not published, leading to transparency and corruption concerns. The manual, inefficient asset-tracking system and the lack of reconciliation between the payroll system and the human resources database were highlighted. The system is fragmented internally between various government entities and further complicated by the use of parallel systems by partners, who have multiple financial management models, some of which are not reported on to the sector. Based on a personal communication with a key government member responsible for PFM at the Federal Ministry of Health in 2022, we found that a PFM improvement plan had been drafted. However, due to frequent changes to the political context, it had not been implemented, and its progress as well as the progress of the partners’ commitments had not been monitored.

The establishment of the National Medical Supplies Fund (NMSF) by law in 2015 has led to a positive change to the availability of medicines and health supplies [22]. This unified government entity has brought together all states of Sudan (except Khartoum) as well as the army and police health services, leading to a reduction of procurement costs and unification of prices. The NMSF procures an extensive list of critical health items and has distribution branches throughout the states it covers [26]. The NMSF aims to harmonize purchasing arrangements across public entities, reduce duplicate procurement channels, and gradually encourage partners to procure medicines and supplies through it. Nevertheless, the availability of supplies is frequently affected by economic constraints such as the availability of foreign currency and political incidents such as port strikes and conflicts. Various assessments have also pointed to gaps in the capacity to estimate the quantities of medicines needed and to distribute these medicines [23,27].

An assessment of the Sudanese health information system commended the effort to have an integrated system through the utilisation of the District Health Information Software Version 2 (DHIS2) platform [28]. However, there are still multiple vertical parallel systems for routine health information, stemming from donor-supported programmes for immunisation, HIV/AIDS, and tuberculosis. These systems are mostly paper based up to higher administrative levels and barely use simple spreadsheets to compile data. Furthermore, the system is plagued by uncoordinated population-based surveys, which lack standardised planning and implementation. The Sudanese health system has poor coverage of death registration and cause of death reporting and has major infrastructural issues at the lower levels, with weak analytical capacities across levels. Efforts to compile data and to ensure that different data, including surveillance of health emergencies, feed into DHIS2 have faced resistance from programme-specific local staff, implementation partners in the field, grants managers at the national level, and large donors [28].

### Data collection and respondents

This study adopted a qualitative case study design to explore the study topic in depth. It adhered to the Standards for Reporting Qualitative Research (SRQR) [29]. Respondents were purposefully defined through engagement with key actors in strengthening the health sector system and partnerships at the Federal Ministry of Health and the WHO, and then through a snowball process, ensuring the relevant partner groups were represented.

A semi-structured interview guide was developed by the researchers based on the study objectives. This guide was frequently amended during the study to incorporate any emerging vital issues. The guide is included in S1 Appendix.

Data were collected through two rounds of interviews. The first round took place in 2015 (between 12 July and 20 August), just a year after the Sudan Health Compact was signed. The second round took place between 16 May 2021 and 11 May 2022, following the revolution and major changes in the partnership scene in the country. From the initial list of targeted respondents in 2015, we identified 24 senior technical staff, preferably at the national level, who were directly involved in health sector partnerships and health system deliberations. We contacted each of them but did not receive a response from two government workers and two INGOs respondents. In addition, one donor declined to participate, and two other donors and a potential government respondent replied late in the process after we had reached data saturation with a total of 16 interviews.

For the second round of interviews, we initially contacted the respondents from the first round if they were still working in the Sudanese health sector. Then we continued with snowball sampling, identifying additional potential respondents as per the recommendations of those we interviewed who identified them as relevant to the discussions and efforts of health system strengthening and partnerships, always ensuring there was a balanced representation of the partner groups. Saturation was reached after 15 interviews. No respondents rejected the interviews invite during this round.

The respondents in both rounds represented diverse partner groups, including government, donors and development banks, UN agencies, INGOs, and national civil society. Four respondents participated in both rounds. Table 1 presents the number of respondents per partner group per round.

**Table 1:** The number of respondents per partner group per interview round.

Partner group	Round 1 (2015) (N = 16 respondents)	Round 2 (2021/22) (N = 15 respondents)
Government	2	4
Donors and development banks	5	3
United Nations agencies	4	3
International non-governmental organisations	2	3
Civil-society organisations	3	2

The majority of the interviews during the second round were conducted online. All interviews were carried out in the respondents’ natural work setting and were conducted in English, a language all respondents were comfortable using. While the private sector is a major partner group, the Sudan Health Compact did not involve any private sector representation, so we did not include them in the present study.

We also reviewed documents for data triangulation. The documents included relevant reports, assessments, and information found online or referred to by the respondents.

### Data analysis

**Fig 1:**
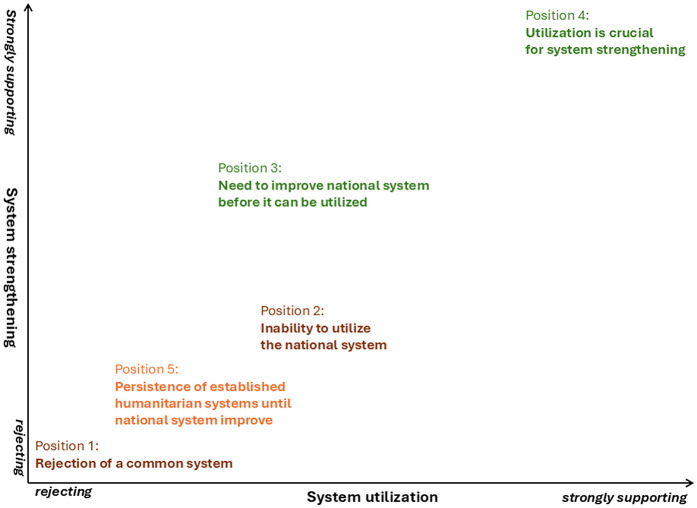
Positional map of partners’ system strengthening and utilisation attitudes and efforts. Brown: position from 2015. Orange: position from 2021/22. Green: position from both rounds.

This study applied situational analysis techniques with positional mapping [30,31]. Interview recordings were transcribed verbatim, and the transcripts were read thoroughly. Then, an abductive coding process of all the transcripts was performed. The open codes that initially emerged from the transcripts were reviewed, with constant comparison to the original text. Codes containing similar ideas regarding system utilisation or strengthening were grouped together into categories. These were again revised against a matrix containing summarised texts of the respondents’ inputs on the system components and their status, strengthening efforts, and positions on utilisation, to ensure the accuracy of the emerging categories. Finally, a positional map was constructed as a visual representation of these final categories. This process was first done separately for the data from 2015 and from 2021/2022, leading to one map for each dataset. In this study, ‘positions’ refer to analytically derived viewpoints regarding system strengthening and utilisation and is the main analytical term. Attitudes or stances refer to respondents’ expressed views, while ‘practices’ refer to actions and implementation behaviours reported by respondents. Practices were analysed as manifestations, justifications, or consequences of these positions. The positions from the two maps and the specific inputs of partners within the two datasets were compared, allowing the two maps to be merged into one (Fig 1), depicting both common positions and positions that were found within only one dataset. This map shows the constant positions, attitudes and practices as well as the changes over time. The comparison was intended to identify continuity and differences in the positions represented across the two study periods rather than to assess attitudinal change among the same individuals, as only four respondents participated in both rounds.

The map comprises two axes: the x-axis captures positions on system utilisation, and the y-axis captures positions on system strengthening. The positions within the map depict the respondents’ input from rejection to a strong support. For example, the scenario where a partner strongly supports utilising the national system can be seen with a statement such as ‘We contracted NMSF for our supported facilities’. The statement ‘we started supporting the national system so that we can be able to use it in the future’ is seen as supporting strengthening the health system and being open to utilising it, but with reservations that were explored further. The statement ‘We don’t follow this common system idea’ is considered a rejection of both strengthening and utilisation. The positions are supported by explanations and quotes.

### Trustworthiness of the study

The interview guide was developed through a rigorous review of the literature and the documents outlining the partner commitments [9,10]. It was modified during the data collection to enable further discovery of areas that emerged from the initial interviews, as advised by Dahlgren et al. [32]. The first author – who had been employed by the Government of Sudan and later by a UN agency, with a function directly related to the study topic – had prior understanding of the local context and a relationship with many of the actors who facilitated the recruitment of high-level respondents. These relationships built their trust in the study. The other authors had had no direct exposure to the Sudanese health system and its partners, so they provided an external view. Credibility, especially grounding the analysis in the data as much as possible, was ensured by developing a matrix including a summary of the respondents’ statements during coding and grouping. Multiple briefings and discussions within the team were carried out throughout the process. The use of the quotes to explain each position and the review of relevant documents helped to ensure the accuracy of the data and the analysis. Providing detailed contextual descriptions is intended to support the credibility of the findings.

### Ethics statement

In 2015, an administrative letter was issued from the designated directorate at the Ministry of Health for data collected as part of the first author’s master’s thesis. Ethical clearance was obtained from the Sudan Ministry of Health Research Ethical Committee in February 2021 for the second round of data collection. The clearance was for the first author’s doctoral study, titled initially ‘Sudan Health Sector Partners Relations, Perspectives and Adherence to the Effective Development Cooperation Principles’, which includes this article among others. This is a continuation of the first author’s master’s thesis with follow-up over time, as clarified in the approved ethical application.

Information about the study was shared with the targeted respondents. Each interviewed respondent provided written informed consent prior to the interview and oral informed consent at the start of the interview. The respondents were assured that their data would be kept confidential and requested to permit the recording of the interviews; they all agreed. Data including interview recordings and transcripts have been stored securely, and the presented results do not include details that can reveal the respondents’ identities.

## Results

The results are highlighted within a positional map (Fig 1). The horizontal (x) axis represents positions regarding utilisation of national health system components, ranging from rejection or non-use at the left end to strong support and active utilisation at the right end. The vertical (y) axis represents positions regarding system strengthening, ranging from rejection or lack of support for strengthening at the bottom to strong support for strengthening at the top. Positions located nearer the upper-right corner therefore indicate stronger support for both strengthening and utilisation. The colours explain whether the specific position is found within both data collection rounds (2015 and 2021/22) or during just one round. The numbering of the positions follows a chronological order.

There were only two positions in 2015: rejection of a common system’ and ‘inability to utilise the national system’. These positions disappeared in 2021/22, when a new position took place, namely ‘persistence of established humanitarian systems until the national system improves’. Further, two positions were present at both time points: the ‘need to improve the national system before it can be utilised’ and ‘utilisation is crucial for system strengthening’. Although these positions summarise the findings, there are different reasonings and dynamics within each position. The main stance within each position is reflected in the main theme while the details (both commonalities and differences) within the position are clarified with quotations that reflect the specific position(s). For each position, we identified the primary partner groups involved and examined how the positions represented among respondents differed between the two study periods.

### Position 1: Rejection of a common system

This position reflects the opinion that there is no need for a unified system for financial management and procurement and supply. This position, found only in the 2015 data collection round, included variety respondents from INGOs and UN agencies as well as a donor. At that time, the government had advocated for unifying all funding procedures by putting all external funds into one basket before making decisions on implementation. The partners who held this position rejected this proposition, arguing that it would cause unnecessary duplication of processes, especially for remote places where the government cannot access or provide services.

*For me, there is no reason to have [a] unified system. We discussed [basket financing] for some time but [it] did not reach anywhere. I don’t think it will be feasible now, because why do we put other layers? This way things go to the government and from the government to the NGOs, national or international, as they are the ones who implement anyway in many areas. Now things go to NGOs anyway, so why do we need to put a government layer?* (INGO 1 – 2015)

These respondents argued that the national financial management system was too weak to handle the processes required. They also noted that some partners could not invest in improving the system, either because they were too small or because such reforms fell outside their mandate. They had had bad experiences when implementation and reporting requirements were not met – including corruption, misuse, and low implementation capacity – leading to potential loss of funds or the need to return funds.

*Our preference is not to go through any government system or public sector, especially for funding, mainly because reporting requirements are not met. There is suspicion of funds misuse, corruption. We are small compared to other UN agencies, and our funds are much smaller. We don’t feel it will make a difference. Others are working in health system strengthening, financial management system strengthening, procurement and supply chain management, but we are not”* (UN agency 3 – 2015)

Some respondents also opposed unifying the procurement and supply system, citing similar concerns about trust and capacity. They argued that their own rules and systems needed to be followed to ensure quality.

*We don’t follow this common system idea. Right now, we have our own procurement rules. We know the Sudanese procurement rules as all procurement should be through the central medical supplies, but [our] government put rules for us that we need to compare and check also private companies and compare the prices and the quality to find the best choices. We follow these rules*. (Donor 3 – 2015)

### Position 2: Inability to utilise the national system

This position includes statements from respondents who had reasons to not use the national system components. Although they did not completely reject the idea of a common system, they had not yet taken steps to use or strengthen its components for various reasons. This position was mainly taken by respondents from CSOs and a donor from the 2015 data collection round.

The respondents from CSOs mentioned a problem with the government not enabling them to utilise the national system for procurement and supply. This was interesting because, unlike other partners who were encouraged by the government to use the NMSF, these partners were not shown the same engagement interest (as depicted in position 4).

*As civil society we can’t use the national system because the government [doesn’t] give that space. The government [doesn’t] enable us to use their procurement and supplies system, for example, like they are trying to with big donors and UN agencies*. (CSO 3 – 2015)

Although the donor within this position was eager to use the health system components, political considerations prevented engagement with the national system. In 2015, the government had major disputes with several Western partners due to financial sanctions and an open ICC case, which blocked Sudan from entering into partnership agreements that conflicted with the ICC rules.

*We cannot use the country[’s] system because Sudan did not sign the Cotonou agreement. The rules prevent us from using the national system. The Government will not sign the agreement due to legal complications with the ICC and political consequences of its actions and global relations.* (Donor 1 – 2015)

### Position 3: Need to improve national system before it can be utilised

This position captures views that the system is weak and has numerous issues that must be fixed, so efforts should first focus on strengthening the national system before utilising it. In 2015, this position was primarily held by respondents from UN agencies and development banks, and by some donors. By 2021/22, it was held by a donor, a respondent from an INGO, and a government representative.

*Currently, [we] cannot use these national system components as the system is very weak.* (UN agency 4 – 2015)

While the respondents who held this position agreed on the importance of building a stronger system, they acknowledged the limitations of the national system, causing trust concerns.

*[The] Sudan[ese] system lacks transparency and accountability. There are issues related to budget adoption and the distribution of funds to different sectors including the security sector; these are the issues that as donors we look at. Parts of our programmes and funds were to provide technical assistance as we support the Ministry of Health to strengthen the system and improve the public financial management.* (Donor 2 – 2015)

In 2015, the majority of donors and respondents from UN agencies stated that they were focused on assessing gaps in the system. They aimed to identify weaknesses so that they could be strengthened before moving directly to system utilisation.

*First, we have to do a macro-assessment and then a micro-assessment to come up with the risks and the gaps, what needs to be strengthened, before you work with the national system.* (UN agency 1 – 2015)

At that time, there were ongoing efforts to strengthen procurement and supply, financial management, and health information. The hope was that these efforts would promote discussions and lead to long-term productivity.

*We supported the government in developing the procurement reforms up to the development of the new procurement law in 2010. System strengthening is [a] long process [before the system can be] utilise[d].*‘. (Development bank 2 – 2015)

However, some partners were piloting utilisation with specific components that they had already assessed and found to meet their standards. For example, regarding financial management functions, some UN agencies applied what is called a harmonised cash transfer (HACT) approach through which the government received grants and funds, and then managed them and reported on their use to these UN agencies. Nevertheless, these agencies reserved the right to do spot checks and audits to ensure everything was going well as per the agreed standards.

*Since 2014 we have been trying, with the government, to implement this ‘HACT’ approach … that is supposed to be the approach for the agencies of UNDP, WFP, UNFPA and UNICEF… with this HACT the idea is that we trust the national financial management system; they don’t have to send us all the documents. They keep the receipts, but occasionally we will come to do a spot check.* (UN agency 1 – 2015)

The adoption of the HDPNx approach in the health sector in Sudan in 2017 seems to have positively influenced the attitudes and actions of INGOs regarding utilisation and strengthening of the national system in 2021/22.

*For medical supplies, certainly we have parallel systems. This is due to many reasons, but the main [one] is the donors funding our programme not having enough trust in the Sudanese medical supplies system to ensure the timely availability of drugs. Yet, as we are piloting this nexus approach to build the capacity of the state and locality level authorities, we had to start supporting the national system so that we can be able to use it in the future.* (INGO 1 – 2021/22)

The donor who in 2015 claimed that the national system could not be utilised changed their position in 2021/22, to a view of the need to strengthen the system to enable later utilisation. Although this donor recognised weaknesses in the system, they noted improvements in transparency and trust, which the respondent associated with greater openness to collaboration following the end of the regime subject to ICC warrants.

*We cannot rely on the government system and mechanisms currently due to their weaknesses. Meanwhile we are supporting strengthening efforts. Currently as we support improving the integrated health information system capacity, we feel the produced reports are more trusted and transparent to build on.* (Donor 2 – 2021/22)

One government respondent in 2021/22 pointed to strengthening and reform of the national system as a reasonable prerequisite before all partners could utilise the system. The respondent stated that utilisation should occur once the system functioned better. This view was different from the other government respondents interviewed during 2015 and 2021/22, who strongly saw system utilisation and strengthening as activities that must go hand in hand.

*System utilisation depends on reforming and strengthening it. The challenges of health information, procurement and supply, and the financial management are huge and most of them are related to the capacities, because the long drainage of good staff through migration and the turnover process. Also, corruption concerns are there. The system needs real reform to rebuild trust.* (Government 2 – 2021/22)

### Position 4: Utilisation is crucial for system strengthening

This position in the top-right corner of the map stems from a belief that without utilising the system, it will not be possible to identify all weaknesses and work on improving them. Moreover, waiting for the system to be perfect would just delay the commitment to unify the health system components as agreed upon in the Sudan Health Compact. This position was present during both data collection rounds.

*When we talk about the system, improving is by utilising it. We can’t put it in shelves and assess and design and later say look it is now beautiful, come take and use it.* (Government 2 – 2015)

Since 2015, the government has actively worked to convince some other partners strengthen the components of the national system and to assess and gradually utilise them. This included convincing other relevant governmental entities and major global partners to lead by example.

*The government managed to establish a unified system for procurement and supply of medicines, a platform among various government entities [such] as the ministry of health, police, army and security medical services, health insurance, and all relevant governmental entities. All now do their procurements through the central medical supply fund. I think this will give us the convincing power that we have one system that all the partners can work through* (Government 1 – 2015)

These efforts have succeeded in bringing major partners to pilot the utilisation of key system components. This approach has allowed the identification of gaps and subsequent improvements that can strengthen the government’s ability to advocate that the national system should be utilised more broadly.

*We started with some partners, for example, with GAVI we convinced them to use our financial management system, so now there is an experience with them as one of the main international donors in health using the government system. This gave us the opportunity to test our system to make sure that it really addresses the needs of our partners. Hence, we will be in a position to say to others that we have a system that will help you and address the agreed needs* (Government 2 – 2015)

While only respondents from the government took this position in 2015, by 2021/22 it was shared by donors and respondents from UN agencies, INGOs, and the government. There had been additional efforts to convince international partners to simultaneously strengthen the components of the national system and utilise them. The respondents reported that this approach had led to notable successes.

*We should all use the system, understand its flaws, and then support it further to overcome them. We are progressing. Lately, following on GAVI steps, Global Fund agreed to invest in the HIS after a detailed survey about its gaps. New projects like those from the World Bank use the Public Financial Management System and the Medical Supplies Fund.* (Government 1 – 2021/22)

The commitment to use the national system while strengthening its capacity was further reinforced during the transitional period (2019–2021), particularly for COVID-19 supplies, when many donors and development banks utilised the NMSF. Overall, donors and respondents from UN agencies appeared to increasingly adopt the position of utilising a unified national system while contributing to its capacity building.

*We have to move away from parallel systems. We are trying to build and support [the] Sudan[ese] system. We aim within the supply of medicines to strengthen the National Medical Supplies Fund.* (Donor 3 – 2021/22)

Progress in the attitudes of donors and respondents from UN agencies towards system utilisation was particularly evident for the health information and procurement and supply components.

*We cannot rely on kits procured through humanitarian operations. Even these should be merged into the national system. Further, for the information [from] all sources should fall into the DHIS 2. We are working on including surveillance components that were managed separately [in] the DHIS2.* (UN agency 2 – 2021/22)

The emergence of INGOs actively using and supporting the national system was striking. Some began to trust the NMSF to supply medicines to their health facilities and to contribute to strengthening its overall capacity.

*We are supporting the local actors and government entities. For example, we brought NMSF to provide drugs and supplies, building drugstores and work in our supported facilities where they were not normally operational.* (INGO 2 – 2021/22)

### Position 5: Persistence of established humanitarian systems until the national system improves

This position was taken only by respondents working in the humanitarian sphere (UN agencies, INGOs and CSOs) during the 2021/22 data collection round. They believed that the national system was weak and unable to deliver the functions the partners needed, so their parallel systems should be maintained. Despite years of investment, they noted that capacity gaps persisted due to political instability and economic constraints and argued that further strengthening should be the responsibility of the government and development partners.

*The national system is weak, even after years of external support, due to political and economic issues. Hence, I cannot agree on national system use as necessity. If we crossmatch what our emergency partners systems do, and the national system, they aim to give same results. As long as my system can function better than the national system then I will keep using them.* (Humanitarian, UN agency – 2021/22)

## Discussion

We have presented a case illustrating the different positions adopted by health sector partners regarding the strengthening and utilisation of the health system components, together with their practices reported in relation to those positions, the drivers and explanations behind their positions, and how these have changed over time. The Sudanese case can provide lessons for global health partnerships and health system strengthening and beyond. In this discussion, generalisations about health systems builds on finding focused on the three studied components: financial management, procurement and supply, and health information.

### Changes in partners’ positions and variations across health system components

There were important differences in the positions represented between 2015 and 2021/22. In 2015, respondents from various partners rejected the need to strengthen or utilise a common national system, while others stated they were prevented from using the national system. However, these two positions were not present during 2021/22. By 2021/22, only some respondents from the humanitarian sphere were resistant to system utilisation, claiming that until the national system improves, they would keep using their functional systems. This position includes a passive stance where the responsibility is placed on others (the development partners).

Among the components, there was mostly rejection of, prevention from, or resistance to utilisation of financial management and to a lesser extent procurement and supplies. Meanwhile, all partners showed interest in having a common health information system, although they expressed differences regarding what variation of efforts would be required to strengthen it. This progressive support for system strengthening and utilisation has been reported by the Health Data Collaborative (HDC), an initiative that convenes stakeholders across the health data ecosystem to strengthen countries’ health information systems. These efforts have been reported in comparable country contexts like Zambia, Cameroon, Kenya, Nepal, and Bangladesh, where policy, financial and operational alignment efforts have advanced [33].

The position emphasising the importance of using the health system while strengthening it was present at both time points. In 2015, it was held solely by respondents from the government, whereas by 2021/22 it was shared by donors and respondents from UN agencies and one INGO. Interestingly, a government respondent from the 2021/22 data collection round stated that strengthening was a prerequisite for utilisation. This view indicates that the government was no longer collectively taking the position of strongly pushing towards further utilisation as in 2015. This shift can be explained in our view by the political changes from a central controlling government to the broad alliance transitional government, perhaps due to the lack of a unified vision during the transitional period (2019–2021), as outlined by Aweesha et al. [19]. They clarified how openness in collaborating with the government was seen during the transitional period, which explains the donors’ movement from the positions of rejection or inability to utilise the system or demanding a strengthened system first in 2015, to engaged stances of actively working on strengthening the system and concurrently utilising it.

Respondents frequently linked these differences in positions to political shifts, mainly the end of the old regime with its constraining warrants from the ICC blocking it from some collaboration agreements, or uprising approaches such as the HDPNx, and the willingness to advance collaboration arrangements with the transitional government. The collective progress of UN agencies into utilising the system can be attributed to their cumulative piloting experience, assessments, and strengthening efforts. Apart from a humanitarian-focused INGO that took the former position of utilization rejection, compared with 2015, INGO respondents in 2021/22 described actively working on strengthening and piloting system utilisation, to even some engaged in utilising and strengthening simultaneously. This could be explained by application of the HDPNx approach to their intervention. By 2021/22, respondents from the national CSOs no longer complained of being prevented from utilising the system, as in 2015. This could be due to their position (as mainly humanitarian actors) who are no longer prevented but will continue to utilise their own systems.

In general, respondents frequently described political context as a central factor shaping relationships among partners and influencing willingness to engage with national systems as well as their uptake of collaboration approaches like the nexus. Those factors were more frequently referred to by the respondents rather than the underlying health system status and needs or the signed partners commitments within the Sudan Health Compact when explaining their positions. This finding is consistent with comparable results reflecting that partners’ practices are often driven by political factors and interests [19,34]. While respondents frequently framed their positions in terms of trust, transparency, and political relationships, these concerns may also reflect broader institutional constraints. Several respondents referred to reporting requirements, corruption concerns, audit expectations, and restrictions affecting engagement with government systems. Such considerations are consistent with donor accountability frameworks and fiduciary risk management requirements that can discourage direct utilisation of national systems, particularly in fragile and politically complex contexts. These structural factors may therefore interact with perceptions of trust and legitimacy when shaping partners’ decisions, however, were not directly evident within our data.

Although the findings suggest increased support for system utilisation by 2021/22, several tensions identified in the study remained unresolved, including concerns regarding system capacity, accountability, and the continued use of parallel systems by some humanitarian actors. Viewed retrospectively in light of the 2023 conflict, the findings may indicate that progress towards greater system utilisation was both dependent on a favorable political environment and constrained by structural challenges that had not been fully addressed.

### The role and relationships of humanitarian partners in the health system

While both humanitarian and development partners operate within settings like the Sudanese health sector, humanitarian partners were more reluctant to work with a unified system, citing system weakness as the key reason. This is in line with findings from other studies where humanitarian NGOs struggle with weak government structures and administrative bureaucracy [35]. Humanitarian and development partners classically engage through different implementation modalities, and their perceptions and expectations about their respective roles differ substantially. In this study, respondents from humanitarian background strongly claimed that they are not responsible for system strengthening. They perceived system strengthening as the role of development partners. They argued that humanitarian partners are always expected to show quick gains and results with tight funding schedules and to act neutrally and impartially differentiating them from development partners who are expected to engage, align, and harmonise with national systems. Hence, humanitarian partners frequently prioritise operational autonomy and maintenance of parallel systems even when there are efforts to strengthen the national system.

This rejection of any role in system strengthening contradicts calls for donors, INGOs, and NGOs to support the recipient countries to rebuild their health systems so that they are resilient and support the achievement of health goals [36,37]. We speculate that this rejection and its reasons intensified following the April 2023 war, with partners hesitant to invest in a system facing instability and major humanitarian needs, consistent with behaviour reported by Aweesha et al. [19]. Even before the first round of data collection for this study, some humanitarian NGOs expressed resistance to integrating within established health service provision models in Sudan [38]. They tended to choose direct service provision models. Although both groups questioned the capacity and reliability of the Sudanese health system, only humanitarian partners persisted in resisting utilisation, framing system strengthening as outside their responsibility and incompatible with emergency imperatives. Nevertheless, it is important to note that some of the respondents from humanitarian partners gradually shifted towards joint strengthening and increased system utilisation by 2021/22, influenced by adoption of the HDPNx. Aweesha et al. [39] argued that the adoption of this approach in Sudan was necessary given the country’s context of protracted emergencies and humanitarian needs with decreasing humanitarian funds.

While humanitarian partners, especially INGOs, fill gaps, there are many concerns regarding their relationships with the public systems within the countries they assist [40]. Pfeiffer et al. [41] proposed a code of conduct for NGOs to act on strengthening health systems in poor countries. It includes elements to ensure system sustainability, including long-term hiring and compensation practices, capacity building, and joint planning. However, the clarity regarding the strengthening and utilisation of health system components – reflected in the IHP+ seven behaviours for development partners – is lacking in humanitarian discussions. This gap should be addressed within the broader framework of humanitarian principles.

### Strengthen first or utilise and improve simultaneously?

Calls to utilise national systems while simultaneously strengthening them are based on the assumption that this approach accelerates the identification of system weaknesses and supports continuous improvement. However, critics argue that it may compel partners to rely on inefficient systems, potentially compromising short-term outcomes. Conversely, delaying system utilisation until improvements are completed can weaken partner commitment, slow engagement, and reinforce reliance on parallel systems perceived as more efficient.

Although it may seem reasonable to require national systems to meet certain standards before partners adopt them – particularly to ensure efficiency and to reduce corruption risks – evidence suggests that maintaining parallel systems creates persistent challenges. These include resistance to national ownership, weak integration into national information and accountability frameworks, and difficulties in transitioning funding and service management [42].

Strengthening efforts should therefore be closely monitored, with clear partner commitments tied to defined milestones, ensuring the gradual and timely phase-out of parallel systems. Early adoption of improved health system components by some partners should be encouraged and used to promote broader alignment. At the same time, the central leadership role of the national government must be sustained, supported by a shared vision across sector leaders. Governments should also enable all partners, including national CSOs, to assess, support, and utilise national health system components.

This study did not assess the comparative effectiveness of these competing governance logics or their impact on system performance; rather, it reflected on how different actors justified and negotiated them within the Sudanese health sector.

### Limitations

There are several limitations to this study. First, despite efforts to maximise the number of respondents and to achieve saturation with reasonable variation across the partner groups, there was a relatively small number of respondents from certain groups, particularly INGOs in 2015 and CSOs in 2021. This may have limited the representation of humanitarian perspectives, as these groups comprise a substantial proportion of humanitarian partners. A similar concern applies to the government respondents in 2015; however, the two respondents included at that time were highly relevant to this study due to their positions.

Second, the interviews conducted in 2022, following the October 2021 coup, were subject to potential recall bias, as the respondents were asked to reflect on the transitional period (2019–2021). The significant political changes, and the resulting shifts in relationships between partners and the government, may have influenced the respondents’ recollections and interpretations. In addition, only four respondents participated in both rounds of data collection. Consequently, the study is not designed to assess longitudinal attitudinal change among the same individuals. Rather, it compares the positions represented among health sector actors at two different time points. High personnel turnover between 2015 and 2021 may have influenced the accuracy of historical accounts and representation of true attitude shifts. Reliance on interviews with senior-level personnel may also have limited the capture of frontline perspectives.

Finally, this study was completed prior to the April 2023 conflict and therefore does not account for subsequent disruptions, changes in partner roles, or shifts in health system capacity resulting from the ongoing crisis. The study cannot determine whether the observed changes represented durable institutional transformation or a temporary period of increased alignment among partners. Future research should examine how conflict affects the sustainability and reversibility of efforts to strengthen and utilise national systems.

## Conclusion

Between 2015 and 2021/22, positions of health sector partners in Sudan shifted from resisting the strengthening and utilisation of a national system to more actively supporting a unified national health system and reducing parallel, agency-led structures. The findings suggest this positions shift was highly shaped by changing political dynamics following the 2019 revolution, as well as by the growing need for resilient approaches, such as the HDPNx, in response to recurrent health emergencies and an increasingly volatile context. Early government-led efforts to pilot the utilisation of a national system helped build trust among some partners, encouraging wider adoption of system-based approaches. Nevertheless, a central tension remains: whether system strengthening should precede system utilisation, or whether utilisation itself is necessary to drive effective strengthening. Resistance among some humanitarian partners to phasing out parallel systems also persists.

Building on these findings, we call for more systematic tracking of partners’ contributions to health system strengthening and to regularly review their commitments to using national systems within existing development cooperation frameworks. Clear milestones should be established and revisited to support the gradual transition away from parallel systems. Documenting and promoting successful examples of system utilisation can further encourage alignment by demonstrating feasibility and building confidence. Strong government leadership remains essential and should be supported by a shared vision across stakeholders. Governments should also enable all partners, including CSOs, to engage with and utilise national health system components. At the same time, clearer guidance is needed on the role of humanitarian partners in both strengthening and using national systems, including how they can better align with national priorities and mechanisms. Donors can support this process by providing flexible funding and policy frameworks.

The subsequent outbreak of nationwide conflict in 2023 further underscores the importance and relevance of understanding the durability of system strengthening and utilization gains within such context where periods of progress may remain vulnerable to reversal.

Finally, while context should always inform the design of health system strengthening efforts, this case study suggests that political dynamics and contextual shifts played a more decisive role than formal agreements in shaping partners’ behaviour under the Sudan Health Compact. This underscores the importance of cooperation frameworks that explicitly account for political realities and volatility.

## Supporting information

S1 Appendix Interview guide. Semi-structured interview guide used to collect the data that explored the attitudes and practices of Sudan’s health sector partners towards strengthening and utilizing national health system components, including financial management, procurement and supply, and health information systems.
(DOCX)

S1 Checklist Inclusivity in Global Research Questionnaire. Completed PLOS inclusivity in global research questionnaire describing the ethical, cultural, and scientific considerations relevant to the conduct of this study.
(DOCX)
