# Supplementary material for: Attitudes and practices of Sudan’s health sector partners towards strengthening and utilising the national health system components (2015–2022)
Source: PLOS Glob Public Health. 2026 Aug 18;6(8):e0007039. doi: 10.1371/journal.pgph.0007039 (PMC13484957; doi:10.1371/journal.pgph.0007039)
Supplement: Supplementary file 1 [file pgph.0007039.s001.docx]

**S1 Appendix**

## Interview guide: Attitudes and practices of Sudan's health sector partners towards strengthening and utilizing national health system components

**Interview No: …**

**Introduction**

There is an international consensus on effective development cooperation principles that can assist in reaching development targets, including those for healthy lives and wellbeing. Sudan is not an exception to these agreements, and a local health compact is in place as well as corresponding strategy. Within this compact the partners have underscored the importance of strengthening the national health system, and gradually but actively utilize key national health system components, namely financial management, procurement and supplies, and the health information component. The aim of this interview is to investigate the attitudes and practices of Sudan's health sector partners towards strengthening and utilizing these national health system components.

**Checklist**

Interview duration: … minutes.

Interview place: ……………………

Request to record the interview: Approved or not

Explanation of how information will be handled with confidentiality: Explained or not

Request for permission to quote: Approved or not

Name of interviewee: **……………………**

Interviewee agency: **……………………..**

Function: **……………………**

Date and time of interview: **……………………**

Written consent: Signed or not

- Questions in red are not to be asked for Government respondents.
- Grey write-up is follow-up questions in case not covered in response.

**Opening questions: Scope of work**

In which capacity and fields do you deal with health sector development cooperation in Sudan? Since when? What have your fields of work been during the last five years? And has this witnessed any changes recently?

**Health system strengthening and utilization**

What is your view on your role in strengthening and utilizing a unified health system? (Refer to specific systems of financial management, procurement and information and take 5 years span.)

Are you now able to use the country systems in delivering your support? (If not ask why. Has this changed?)

What efforts are going to ensure strengthening and utilizing the country’s systems in the cooperation if any? (Refer to specific systems of financial management, procurement and information and take 5 years span.)

Have the health sector partners agreed on a process for harmonized systems for funding modalities, financial management and procurement? If yes, describe it please and the progress on it so far.

Do you share all information on your work with the other sector partners? (If not ask why). In which ways?

Have there been regular joint reviews during the last five years? Elaborate on how it was done or why not.

What are the challenges you face in utilizing the country systems of PFM, procurement and information? Have they changed during last 6 years? Kindly advise on any opportunities you see in this regard.

Are there other factors influencing the implementation of these agreed-upon commitments to align and harmonize the work in the health sector and use national system components discussed? Please elaborate

**Concluding remarks**

Is there anything you want to add to this interview?

Ask if the interviewee knows others who are important to interview. Why and if interviewee can facilitate the process?

Ask for any relevant documents

Thank the respondent
